# SiamHSFT: A Siamese Network-Based Tracker with Hierarchical Sparse Fusion and Transformer for UAV Tracking

**DOI:** 10.3390/s23218666

**Published:** 2023-10-24

**Authors:** Xiuhua Hu, Jing Zhao, Yan Hui, Shuang Li, Shijie You

**Affiliations:** 1 School of Computer Science and Engineering, Xi’an Technological University, Xi’an 710021, China; 2 State and Provincial Joint Engineering Laboratory of Advanced Network, Monitoring and Control, Xi’an 710021, China

**Keywords:** object tracking, Siamese network, feature fusion, transformer architecture

## Abstract

Due to high maneuverability as well as hardware limitations of Unmanned Aerial Vehicle (UAV) platforms, tracking targets in UAV views often encounter challenges such as low resolution, fast motion, and background interference, which make it difficult to strike a compatibility between performance and efficiency. Based on the Siamese network framework, this paper proposes a novel UAV tracking algorithm, SiamHSFT, aiming to achieve a balance between tracking robustness and real-time computation. Firstly, by combining CBAM attention and downward information interaction in the feature enhancement module, the provided method merges high-level and low-level feature maps to prevent the loss of information when dealing with small targets. Secondly, it focuses on both long and short spatial intervals within the affinity in the interlaced sparse attention module, thereby enhancing the utilization of global context and prioritizing crucial information in feature extraction. Lastly, the Transformer’s encoder is optimized with a modulation enhancement layer, which integrates triplet attention to enhance inter-layer dependencies and improve target discrimination. Experimental results demonstrate SiamHSFT’s excellent performance across diverse datasets, including UAV123, UAV20L, UAV123@10fps, and DTB70. Notably, it performs better in fast motion and dynamic blurring scenarios. Meanwhile, it maintains an average tracking speed of 126.7 fps across all datasets, meeting real-time tracking requirements.

## 1. Introduction

Object tracking has been a fundamental and challenging task in computer vision research, remaining a hot topic for decades, with significant progress made in tracking common targets. However, with the rapid development of the UAV industry, UAVs have emerged as promising technology due to their low cost, compact size, and flexible operation. They are increasingly deployed in various applications, such as traffic monitoring, administrative law enforcement, film, and television production, etc. The widespread use of UAVs has led to a substantial increase in UAV image data, prompting researchers to focus on the intelligent processing of UAV images. In particular, single object tracking based on UAV images has gained significant attention. Conventional trackers designed for general targets do not perform well when applied to UAV image tracking. This is primarily due to the unique technical challenges posed by UAV images, including small and fast-moving tracking targets, low-resolution images with high noise levels, and limited computational resources available on UAV devices. As a result, there is a pressing need to develop specialized trackers that can effectively address the specific challenges of tracking small objects in UAV images while operating within the constraints of limited computational resources on UAV devices.

GOTURN [1] was the first high-speed depth tracker, reaching 100 FPS on the GPU. SINT [2] was the first tracker to apply Siamese networks in object tracking. Subsequently, SiamFC [3] creatively employed a fully convolutional Siamese neural network for object tracking, eliminating the need for the tracking search image to match the size of the template image, which allowed the network to utilize a larger search image as input. SiamRPN [4] proposed the deployment of a Region Proposal Network (RPN) integrated with the Siamese network for object tracking. SiamRPN++ [5] solved the problem that the tracker has been using a shallow network (AlexNet [6]) since SiamFC, and the performance decreases when switching to a deeper network. It replaced the less-layered AlexNet network with a deeper ResNet [7] network to extract deeper semantic information, which can significantly improve the network’s capability. TransT [8] combined Transformer, which can effectively establish the association between feature information, with the Siamese network, and proposed a novel tracker that combines the twin framework, an improved Transformer, and a categorical regression header.

However, due to the unique perspective of UAV image tracking, usually the tracked targets are small, necessitating innovative solutions from the trackers designed for UAV tracking. An et al. [9] proposed a novel CF tracking model to learn spatially regularized CF with Hilbert-Schmidt Independence Criterion (HSIC_SRCF) in Reproducing Kernel Hilbert Space (RKHS), which improves the discriminative power by exploiting the changing foreground and background information and adapting to the complex video attributes during tracking. SiamAPN [10] cross-correlated and concatenated the features of the template branch and the search branch hierarchically to achieve feature enhancement. SiamTPN [11] proposed to combine the feature pyramid, which is usually used for small object detection, with the Transformer in a novel way to avoid a large amount of computation and resource consumption while enhancing the features. Hu et al. [12] have employed an integral controller to track and continually update target features in order to ensure that objects captured by the drone, susceptible to deformations and background changes, remain easily identifiable. Zuo et al. [13] proposed a tracking-oriented adversarial blur-deblurring network consisting of a novel deblurrer and a new blur generator, where the deblurrer progressively refines the features through three phases: pixel, spatial, and channel, in order to achieve excellent deblurring performance. Li et al. [14] presented a comprehensive framework that maximizes the utilization of stereo representations in UAV tracking. This approach aims to tackle the challenge faced by state-of-the-art tracking frameworks, which often rely on template matching and struggle with multiple object views in consecutive frames. Furthermore, using a general image-level pre-training backbone may lead to overall representation overfitting, resulting in biases when learning object-level properties in UAV tracking. Additionally, Fu et al. [15] conducted a thorough evaluation and analysis of cutting-edge Siamese network-based trackers.

Currently, available general trackers have demonstrated strong performance in tracking common target tasks but are not applicable to UAV tracking scenarios. Several object tracking methods proposed for UAVs lack the comprehensive consideration of small targets, high ambiguity, and low computation required for UAV target trackers. To address these issues in UAV tracking, we design a feature fusion method that fosters interaction between various levels of features. This method involves the addition of a modulation enhancement layer to the Transformer and the inclusion of a feature sparse enhancement module before the feature map is input into the Transformer. We construct an information interaction fusion module, which integrally uses the last three layers of feature maps extracted from the features to interactively fuse the information of the lower and higher layers in order to solve the problem that the higher-level features tend to ignore the small targets. Furthermore, we optimize the feature sparse enhancement module by employing two sparse attention mechanisms instead of the traditional self-attention. This approach preserves the dense relationships found in the original self-attention without significantly increasing computational demands while enhancing the features.

Overall, the contributions of this work can be summarized as follows:

- A feature enhancement method is proposed. The method divides the features extracted from the backbone network into five layers and interactively fuses the features of the last three layers. Additionally, the incorporation of low-level features effectively addresses the challenge of information loss during the feature extraction process, particularly for small targets.
- A sparse enhancement module is designed. This module interleaves two kinds of sparse attention to replace the original self-attention, enhancing features while avoiding the addition of excessive computation as much as possible.
- Modifications are made to the Transformer. A modulation enhancement layer is added to the encoder of the Transformer to tap the interdependence between features at different levels and enhance them from both channel and spatial directions using triplet attention.
- The proposed method performs state-of-the-art in four air tracking benchmarks UAV123 [16], UAV123 [16], DTB70 [17], and UAV123@10fps [16].

## 2. Related Work

### 2.1. Siamese Network in Tracking

Ever since the initial application of Siamese networks for object tracking in SINT [2] and the first combination of convolutional neural networks with Siamese networks for tracking tasks in SiamFC [3], Siamese networks have gradually demonstrated their immense potential. Subsequent improvements proposed for Siamese network-based trackers were mostly modified in several directions.

For feature extraction: SiamRPN++ [5] modified the backbone network of the original Siamese tracker and replaced AlexNet with the improved ResNet-50 to improve the tracker’s utilization of deep features. SiamDW [18] proposed a unique residual module and used it to develop new architectures with controllable receiver field sizes and network step sizes. SE-SiamFC [19] addressed the issue of target rotational scaling during tracking affecting target localization by modifying the backbone network to use scale-equivariant convolution and scale pooling. This allowed them to record additional scale correlations between multiscale features to gather multivariate information. While it was true that deepening the backbone network could enhance the feature extraction capability of the tracker, it was important to consider computational cost, especially in the context of UAV tracking.

From the perspective of feature refinement, the RASNet [20] superimposed dual and residual attention to capturing both target commonalities and differences, and residual attention can reduce computation by encoding global information about the target. SiamAttn [21] developed a DSA (Deformable Siamese Attention) module, which consisted of two sub-modules, the self-attention module, and the cross-attention module, to enhance the computation of attentional features that improve target-background discriminability. SA-Siam [22] added channel attention to the last two layers of the backbone network to achieve target adaptation. SiamGAT [23] used a graph attention module (GAM) to connect the Siamese backbone to the tracking head and activate the target-aware region so that it can adapt to a variety of aspect ratios that are common in computer vision. SiamBM [24] introduced angle estimation and spatial masking, which effectively enhance features and avoid distraction by cluttered contextual information in the background. DSiam [25] proposed a dynamic Siamese network, which is co-trained with labeled video sequences instead of using only picture pairs in the training phase, giving the network full access to the rich spatiotemporal information of the moving object and learning all parameters offline. UpdateNet [26] introduced the concept of the target template learning to update itself, effectively preventing tracker tracking failures resulting from changes in appearance. GCT [27] suggested the combination of two categories of graph convolutional networks (GCNs [28]), namely, spatiotemporal GCNs and contextual GCNs for graph convolutional tracking (GCT). The simultaneous utilization of spatiotemporal and contextual information can empower the tracker to accurately differentiate between foreground and background elements even in intricate UAV tracking scenarios. While many of the diverse feature enhancement approaches solely exploit the deepest features, consequently enhancing the tracker’s performance, they often overlook the potential of leveraging low-level features.

From other directions: SiamBAN [29] proposed a Siamese box adaptive network that outputs four channels for bounding box estimation in the regression module. The number of output variables is fewer compared to an anchor-based tracker. SiamCAR [30] reconsidered classification and regression methods by viewing the tracker as consisting of two simple sub-networks: a Siamese sub-network for feature extraction and a classification regression sub-network for bounding box prediction. SiamCorners [31] introduced an anchor-free Siamese corner network, where the bounding box estimation of a target can be converted into a pair of corner predictions (lower right and upper left corners). SiamAPN [10] differed from anchor-based and anchor-free methods by building on the notion of anchor suggestions, avoiding the need to pre-define a large number of fixed-size anchors, and achieving better precision through refinement. SiamAPN++ [32] is based on Attention Aggregation Network (AAN) to improve the robustness in handling complex scenes. SiamRPN [4] modified the original RPN by combining RPN with the Siamese network for doing edge prediction and demonstrated good performance. LightTrack [33] had a lightweight design for object tracking. SiamTri [34] introduced a new loss, and so on. While the aforementioned trackers rely on complex designs to achieve high tracking precision, they do not propose solutions for the small tracking targets commonly encountered in UAV tracking missions.

To address the aforementioned shortcomings of existing trackers, this study suggests employing a lightweight backbone network and introducing a feature fusion and sparse enhancement module specifically designed for tracking small targets. These enhancements are aimed at improving the tracking speed of the lightweight tracker while maintaining the precision of the tracking outcomes.

### 2.2. Transformer in Tracking

Transformer is a model that uses an attention mechanism to increase the speed of model training, first proposed by Vaswani et al. [35] for machine translation and later introduced into computer vision and other fields due to its strong performance. It has found numerous applications in the field of tracking. Hu et al. [12] modified the Transformer to utilize self-attention to enhance the features of the template branch and the search branch, respectively and then used a cross-attention-based decoder to feature both branches, highlighting useful global contextual information. Chen et al. [8] designed a feature fusion network using Transformer, where the two branches of the Siamese network are augmented with attention using cross-attention fusion. Attention enhancement is followed by using cross-attention fusion, after which the fused feature vectors are fed into the classification and regression module to get the final target location. The algorithm performs well and is fast. On the other hand, SiamTPN [11] harnessed the inherent feature pyramid of the lightweight network (ShuffleNetV2) and further improved it using the Transformer. This construction aimed to establish a robust target-specific appearance model. Meanwhile, HiFT [36] employed multiple convolutional layers in the similarity computation. However, the network model utilized, AlexNet, was simpler. The Transformer was employed to achieve spatial interaction fusion for shallow convolutional layers and semantic information enhancement for deeper convolutional layers.

In this work, considering that the fast motion of the target is prone to cause dynamic blurring, a modulation enhancement layer has been incorporated into the Transformer’s encoder to fully exploit the dependencies between different layers.

## 3. Methods Proposed

**Figure 1:**
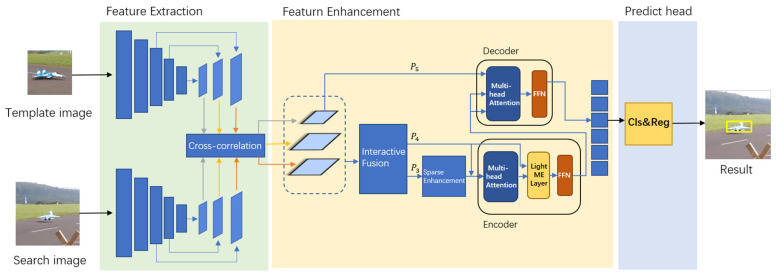
Network structure of the proposed object tracking algorithm.

This section will introduce in detail the proposed tracker: SiamHSFT. As shown in the general framework Figure 1, the tracker of this work is divided into three parts: feature extraction, feature enhancement, and prediction head. The feature extraction part uses a backbone network to extract feature information from the images corresponding to the template branch and the search branch. Feature enhancement is accomplished by an information interactive fusion module, a feature sparse enhancement module, and a hierarchical triple Transformer proposed in this paper. The prediction head performs classification and regression operations on the features that have undergone feature enhancement.

### 3.1. Feature Extraction

The method proposed in this work takes a pair of images (search image and template image) as inputs to the backbone network in the feature extraction phase. Recognizing that UAV-based image tracking tasks cannot afford the heavy computation associated with too deep networks, this work discards the deep network chosen by most trackers nowadays and chooses the lightweight network AlexNet as the backbone network for feature extraction. (Templates and search images are denoted by *Z* and *X*, respectively.) This work divides AlexNet according to each convolutional block as a part, which can be divided into five layers denoted as $L_{i},\;\;i=1,\;2,\;3,\;4,\;5$. The issue of weak extraction power due to using lightweight networks will be compensated by the feature enhancement module.

### 3.2. Feature Enhancement

The feature enhancement part is mainly divided into three steps:

1. Propose an interactive fusion module to fuse the features extracted from the two branches and use them hierarchically.

In order to obtain a more precise feature image, the feature information of the search branch and the template branch is used at the same time. The three layers of feature maps obtained by a correlation operation between the same layers of the last three layers of features extracted by the backbone network will be used in a hierarchical manner by the interactive fusion module. This module has the capability to emphasize detailed information in low-level features as well as semantic information in high-level features.

2. Feature sparse enhancement module is set up after the interactive fusion module.

Based on a kind of interlaced sparse attention, a feature sparse enhancement module is proposed to enhance the features in a way that positively affects the tracking effect with only a small increase in the tracker time complexity as well as in the space complexity.

3. Sparsely enhanced features are hierarchically fed into the improved Transformer for feature fusion.

Fusing features at various levels involves the combination of the modulation enhancement layer. This process strengthens the interconnections between features across different layers, thereby enhancing the target’s discriminative capacity, particularly when motion blur is present.

#### 3.2.1. Information Interactive Fusion Module

**Figure 2:**
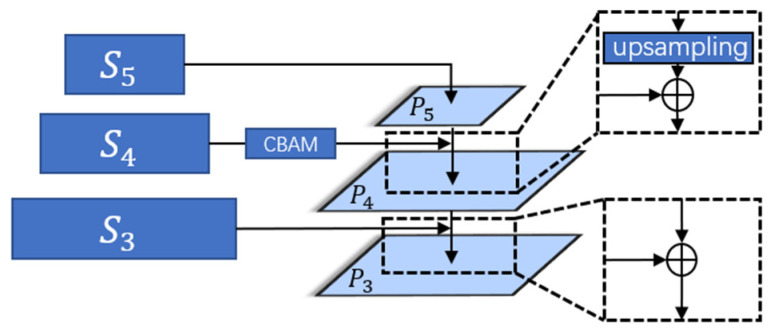
Information interactive fusion module.

As shown in Figure 2, after the features are extracted from the backbone network, this work uses an information interactive fusion module to enhance the template features and search features, respectively, as a way to solve the problem that the high-level features tend to ignore the small targets, and at the same time, to reduce the possible negative impacts on the results from the subsequent sparse operations.

The interactive fusion module retains some of the detailed information contained in the low-level features and fuses them with the high-level features so that the features at different scales contain rich semantic information, which is conducive to accomplishing the small object tracking task in this work. The interleaved fusion module only processes the feature vectors $f_{z}^{3},\;f_{z}^{4},\;f_{z}^{5}\in {\mathbb{R}}^{H_{z}\;\times \;W_{z}\;\times \;C}$ and $f_{x}^{3},\;f_{x}^{4},\;f_{x}^{5}\in {\mathbb{R}}^{H_{x}\;\times \;W_{x\;}\;\times \;C}\;$ corresponding to the last three layers extracted from the backbone network. Before performing the fusion, this work does cross-correlation as well as deformation operations on the last three layers of features of the two branches, which can be expressed as:

(1) $$S_{i}=S\left(f_{z}^{i}\;\times \;f_{x}^{i}\right),i=3,4,5$$

where  ×  denotes the cross-correlation operation and S denotes the deformation operation. To conveniently facilitate subsequent calculations, the channels of the three-layer feature were standardized to 256, $S_{i}\in {\mathbb{R}}^{H\;\times \;W\;\times \;256}$.

The obtained $S_{3},S_{4},S_{5}$ are then subjected to interleaved fusion. Since the fourth of the last three layers to be operated on is the one that best balances the high-level semantic information contained in the features with the low-level detail information, this work enhances the features in this layer so that they first pass through a CBAM [37] attention module consisting of a combination of channel attention and spatial attention.

In order to allow the fusion of two features with different scales, this work has to make the two layers’ feature scales consistent. An upsampling operation is performed on $S_{5}$ to make its scale consistent with $S_{4}$ before allowing the two layers’ features to be summed and fused. And finally, the scale of $P_{i}\in {\mathbb{R}}^{H\;\times \;W\;\times \;d}\;,\;\;i=3,\;4,\;5$ is set by a convolution operation, we employ *d* = 192 in our implementation.

Specifically, it can be expressed as follows:

The fifth layer does not need to be fused, and the convolution operation is performed directly:

(2) $$P_{5}=Conv_{5}\left(S_{5}\right)$$

The result of the fusion of the fourth and fifth layers can be expressed as follows:

(3) $$P_{4}=Conv_{4}\left(CBAM\left(S_{4}\right)+Tran\left(P_{5}\right)\right)$$

where Tran denotes the upsampling operation. In this paper, the transposed convolution operation is used to increase the scale of the fifth layer of the feature map.

Combining the third and fourth layers is a more straightforward process compared to fusing layers 4 and 5. This is because the feature scales of the former two layers match, eliminating the need for an up-sampling operation. The fusion outcome of the third and fourth layers can be expressed as follows:

(4) $$P_{3}=Conv_{3}\left(S_{3}+P_{4}\right)$$

As illustrated above, in this work, we opt for the addition operation to fuse feature maps from different levels, instead of utilizing the concatenate operation. This choice is driven by the consideration that the concatenate operation can inflate the number of channels, resulting in the introduction of superfluous parameters and increased computational complexity.

#### 3.2.2. Feature Sparse Enhancement Module

As shown in Figure 1, before $P_{3}$ enters the hierarchical Transformer as an input, this work augments it again with a feature sparse augmentation module based on interlaced sparse attention.

The traditional self-attention mechanism requires modeling long-distance dependencies by computing the contextual information of each output position and paying attention to all input positions. Traditional self-attention can be expressed as:

(5) $$A=Soft\left(\frac{\theta \left(X\right)\Phi {\left(X\right)}^{T}}{\sqrt{d}}\right)$$

(6) Z=Ag(X)

where X represents the input feature map, A denotes the dense matrix, and Z represents the output features, the self-attention mechanism employs two distinct transformation functions, *θ* and *Φ*. These functions serve to project the input into a lower-dimensional space, and the subsequent self-attention mechanism leverages the inner product within this reduced-dimensional space to compute the dense affinity matrix A. The scale factor, denoted as *d*, is employed in this process. Furthermore, self-attention utilizes the function g to facilitate the learning of improved embeddings. For ease of computation, we assume that the input feature map dimensions are *H × W × C*, using the traditional attention mechanism for feature enhancement, the computational complexity of the self-attention mechanism can be approximated as $O\left(2HWC^{2}+\frac{2}{3}{\left(HW\right)}^{2}C\right)$.

The feature sparse enhancement module designed in this paper optimizes the contextual information computation compared to the traditional self-attention approach. The relationship between all input and output positions of attention in the traditional approach is represented by a dense matrix in which each input position points to all output positions. In contrast, in the interlaced sparse attention used in this work, a dense matrix is split into two sparse matrices: the long-distance attention $A^{L}$ and the short-distance attention $A^{S}$, which are used to attend to long-distance dependencies and short-distance dependencies, respectively. Combining these two attention mechanisms can propagate information from each input location to all output locations, and this interlaced sparse attention complexity can be expressed as $O\left(4HWC^{2}+3{\left(HW\right)}^{\frac{3}{2}}C\right)$ after minimization, which increases the computational amount less than that required by the original self-attention.

Specifically, for long-distance attention, $X^{L}=Permute\left(X\right)$ is first computed by applying a permutation on the input feature map X. Then, $X^{L}$ is divided into *P* partitions, and each partition contains *Q* neighboring positions (*N = P × Q*) as follows: $X^{L}={\left[{X_{1}^{L}}^{⊤},\;{X_{2}^{L}}^{⊤}\;>,\ldots ,\;\;{X_{P}^{L}}^{⊤}\right]}^{⊤}$, where each $X_{p}^{L}$ is a subset of $X^{L}$ with shape ${\mathbb{R}}^{Q\;\times \;C}$. This work applies self-attention independently on each $X_{p}^{L}$, as follows:

(7) $$A_{P}^{L}=Soft\left(\frac{\theta \left(X_{p}^{L}\right)\Phi {\left(X_{p}^{L}\right)}^{T}}{\sqrt{d}}\right)$$

(8) $$Z_{P}^{L}=A_{P}^{L}g\left(X_{p}^{L}\right)$$

where $A_{P}^{L}$*∈*${\mathbb{R}}^{Q\;\times \;Q}$ is a small affinity matrix based on all positions from $X_{p}^{L}$ and $Z_{P}^{L}$*∈*${\mathbb{R}}^{Q\;\times \;C}$ is an updated representation based on $X_{p}^{L}$.

In the end, we merge all the $Z_{P}^{L}$ from different groups and get the output.

(9) $$A^{L}=diag\left(A_{1}^{L}\;,\;A_{2}^{L}\;,\cdots ,A_{P}^{L}\;\right)$$

Similar to long-distance attention, $X^{S}=Permute\left(X^{L}\right)$ in short-distance attention. Divide $X^{S}$ into *Q* partitions, each containing *P* neighboring positions. It is denoted as: $X^{S}={\left[{X_{1}^{S}}^{⊤},\;{X_{2}^{S}}^{⊤}\;>,\ldots ,\;\;{X_{Q}^{S}}^{⊤}\right]}^{⊤}$, after the same operation as in long-distance attention:

(10) $$ISA=A^{S}=diag\left(A_{1}^{S}\;,A_{2}^{S}\;,\;\cdot \;\cdot \;\cdot \;,A_{Q}^{S}\;\right)$$

So, the output of $P_{3}$ after sparse enhancement, the module can be expressed as:

(11) $$P_{3}^{\prime }=ISA\left(P_{3}\right)$$

#### 3.2.3. Hierarchical Triplet Transformer

**Figure 3:**
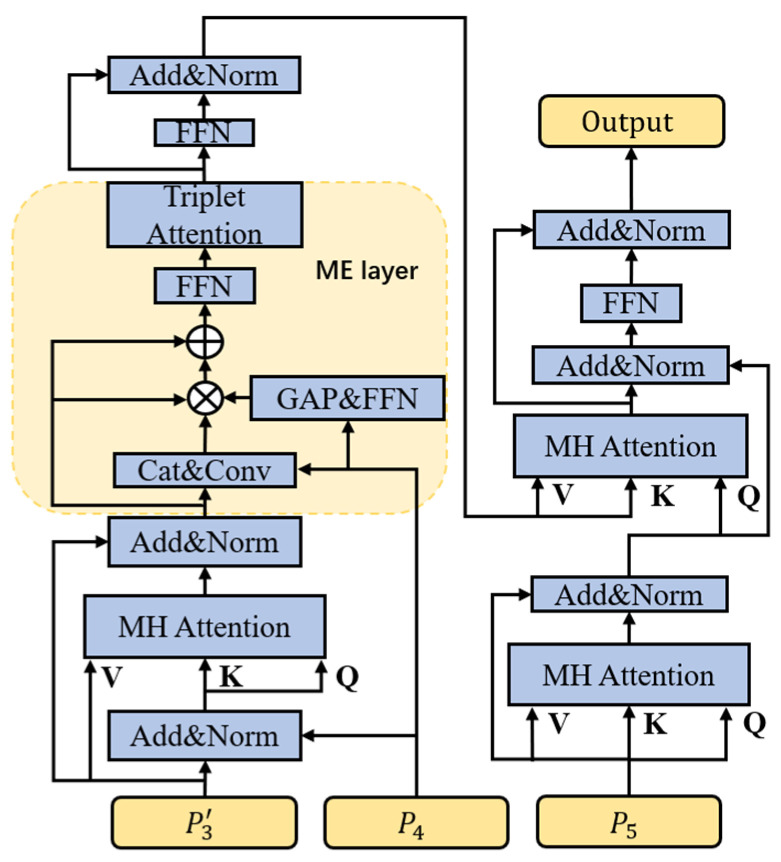
Transformer structure.

Traditional Transformers usually use a feature vector directly as *Q*, *K*, *V* for calculation, which only uses the extracted features of the highest layer and ignores the efficient detail information that is still partially contained in the lower layer features during the extraction process. In tracking tasks, the layer-by-layer extraction of the backbone network may result in the loss of almost all the information about some small objects, and this is where the information contained in the lower layer features becomes particularly important. In the hierarchical triplet Transformer of this work, the inputs of $\;P_{3}^{\prime },\;P_{4},P_{5}$ layered obtained from the previous computation are fed into the Transformer, and $P_{3}^{\prime },P_{4}$ are used as the inputs to the encoder, which is then decoded with the high-level feature $P_{5}$. The detailed structure is shown in Figure 3. This way of hierarchical use of low-level and high-level features by the Transformer can fully utilize the useful information in the low-level and high-level features. In general, this work represents the self-attention mechanism in Transformer partially as:

(12) $$Attention\left(Q,K,V\right)=soft\max\left(\frac{QK^{⊤}}{\sqrt{d}}\right)V$$

where $\sqrt{d}$ is the key element that normalizes the attention. Additionally, the computation of multiple attention can be denoted as:

(13) $$mAtt\left(Q,K,V\right)=Cat\left(H_{1},\cdots ,H_{N}\right)W^{O}$$

(14) $$H_{j}=Attention\left(QW_{j}^{Q},KW_{j}^{K},VW_{j}^{V}\right)$$

where $\;W_{j}^{Q}\in {\mathbb{R}}^{C\;\times \;d_{head}}$, $W_{j}^{K}\in {\mathbb{R}}^{C\;\times \;d_{head}}$, $W_{j}^{V}\in {\mathbb{R}}^{C\;\times \;d_{head}}$, and $W^{O}\in {\mathbb{R}}^{C\;\times \;C}$ ($d_{head}=\frac{C}{N},N$ is the number of heads of the multicentre attention) Cat denotes the concatenation operation. Thus, in the structure designed in this work, the first multi-head attention in the encoder is denoted as:

(15) $$A_{e}^{1}=mAtt\left(N_{e}^{1},N_{e}^{1},P_{4}\right)$$

where $N_{e}^{1}=Norm\left(P_{3}^{\prime }+P_{4}\right)$ and Norm represents the normalization layer.

The modulation enhancement (ME) layer, situated after this multi-head attention process, is used to fully exploit the interdependence that exists between $P_{4}$ and $P_{3}^{\prime }$. The specific operation is shown in Figure 3. the result of the normalization operation between $A_{e}$ and $P_{3}^{\prime }$ is expressed as $N_{e}^{2}$, denoted as $N_{e}^{2}=Norm\left(A_{e}^{1}+P_{3}^{\prime }\right)$. The modulation enhancement module commences with a global average pooling operation and a feed-forward neural network to fuse $P_{4}$ to capture the interdependence between the third and fourth layers aided by the raw information of $P_{4}$. Specifically, this can be expressed as:

(16) $$M^{1}=Conv\left(Cat\left(N_{e}^{2},P_{4}\right)\right)\;\times \;FFN\left(GAP\left(P_{4}\right)\right)$$

(17) $$M^{2}=N_{e}^{2}+\gamma_{1}\;\times \;M^{1}\;\times \;N_{e}^{2}$$

And where $\gamma_{1}$ denotes the weights in a study.

In this work, in order to perform information enhancement of $M^{2}$, a triplet attention is added to strengthen the interdependence between the channel dimension and the spatial dimension by capturing the interaction between the spatial dimension and the input tensor channel dimension. This triplet attention consists of three parallel branches, two of which are responsible for capturing the cross-dimensional interactions between channel C and the space H or W. The last branch is used to construct the spatial tensor. The last branch is used to construct the spatial attention. The outputs of the final tree branches are aggregated using averaging. Inter-dimensional dependencies are established through rotation operations and residual transformations, and inter-channel and spatial information is encoded with negligible computational overhead.

(18) $$A_{e}^{2}=Trip\left(M^{2}\right)$$

Benefiting from the modulation enhancement module, this work exploits the interdependence between $P_{4}$ and $P_{3}^{\prime }$ and enhances both the channel information and the spatial information at the same time with little increase in computational effort, increasing the discriminative power of the target in complex contexts without adversely affecting the lightness of the modeling requirements.

The structure of the decoder is similar to that of the original Transformer, but in order to avoid a direct impact on the transformed features, this work decided to implicitly introduce positional information through the encoder.

### 3.3. Prediction Head

The prediction header’s structure for classification and regression closely resembles that of established sequential network trackers. However, it distinguishes itself from the conventional setup by concurrently employing two classification branches in the classification process. One of these branches is dedicated to classifying the area encapsulated by the ground truth frame, while the other branch’s primary focus lies in identifying positive samples based on the proximity between the center of the ground truth and the corresponding point. The synergy between these two branches is instrumental in enhancing overall accuracy.

Hence, the comprehensive loss function can be defined as:

(19) $$L_{overall}=\lambda_{1}L_{cls1}+\lambda_{2}L_{cls2}+\lambda_{3}L_{loc}$$

In this equation, $L_{cls1}$, $L_{cls2}$, and $L_{loc}$ represent the cross-entropy, binary cross-entropy, and IoU losses, respectively. The coefficients $\lambda_{1}$, $\lambda_{2}$, and $\lambda_{3}$ serve as weighting factors that balance the influence of each loss component.

### 3.4. Algorithm Implementation

This work is based on training with four publicly available large datasets: COCO [38], COT-10K [39], Youtube-BB [40], and ImageNet VID [41]. The images in these datasets are preprocessed, cropped into pairs, and then used as input for the template image and search image, respectively. The backbone network extracts both deep and shallow features from the images and performs interactive enhancement fusion between different layers of the same branch. Additionally, it performs fusion between the corresponding layers of different branches successively. After sparse enhancement and dimensionality adjustment, the fused upper and lower features are fed into the Transformer. The dependencies of the third and fourth features are mined and fused in the Encoder. Subsequently, the output of the Encoder interacts with the fifth feature in the Decoder, achieving the purpose of feature enhancement. Finally, classification and regression are used to determine the target’s location. The entire implementation flow of the proposed method is shown in Algorithm 1.
**Algorithm 1:** Procedure of the proposed method.1.$f_{z}^{3},f_{z}^{4},f_{z}^{5}$ ←  φ(z);# The template is fed into the backbone network AlexNet, and the feature vectors of the last three layers are computed.2.$f_{x}^{3},f_{x}^{4},f_{x}^{5}\;\leftarrow \;\varphi \left(x\right);$# In the search branch, the same process is repeated, and the feature vectors of the last three layers are computed.3.$S_{3},S_{4},S_{5}\;\leftarrow \;S\left(f_{z}^{i}*f_{x}^{i}\right)\;i=3,4,5$;# The feature vectors of the corresponding layers undergo cross-correlation operations to achieve a unified vector size.4.$P_{5}$ ← $\;Conv5\left(S_{5}\right)$;5.$P_{4}$ ← $\;Conv4\left(CBAM(S_{4})+Tran\left(P_{5}\right)\right)$;6.$P_{3}$ ← $Conv3\left(S_{3}+P_{4}\right);$
# Three-layer feature interactive fusion process.7.$P_{3}^{\prime }=ISA\left(P_{3}\right);$# Feed the features to the Sparse attention.8.$Output_{en}$ ← $Encoder\left(P_{3}^{\prime },P_{4}\right)$;$\#\;\mathrm{Feature}\;\mathrm{map}\;P_{3}^{\prime },P_{4}$ input to Encoder.9.$Output_{de}\;\leftarrow$$Decoder\left(Output_{en},P_{5}\right)$;
$\#\;\mathrm{Fuse}\;\mathrm{the}\;\mathrm{Encoder}\;\mathrm{information}\;\mathrm{and}\;\mathrm{the}\;5\mathrm{th}\;\mathrm{Feature}\;\mathrm{in}\;\mathrm{the}\;\mathrm{decoder}.\;\mathrm{Get}\;\mathrm{the}\;\mathrm{final}\;\mathrm{feature}\;\mathrm{map}\;Output_{de}$.10.$B^{*}$ ← $\;\mathrm{Classification}\;(Output_{de}$$)\;\mathrm{and}\;\mathrm{Regression}\;(Output_{de}$);
# Determining the location of targets through classification and regression networks.11.$L_{overall}$ ← $\lambda_{1}L_{cls1}+\lambda_{2}L_{cls2}+\lambda_{3}L_{loc}$;

$\#\;\mathrm{The}\;\mathrm{total}\;\mathrm{loss},\;\mathrm{calculated}\;\mathrm{from}\;\mathrm{cross}\text{-}\mathrm{entropy}\;(L_{cls1}$)$,\;\mathrm{binary}\;\mathrm{cross}\text{-}\mathrm{entropy}\;(L_{cls2}$)$,\;\mathrm{and}\;\mathrm{IoU}\;(L_{loc}$) losses, is used to optimize the tracker.12.until the end of the sequence

## 4. Experimental Results and Analyses

### 4.1. Implementation Details

The model proposed in this work was trained using four large datasets they are COCO, GOT-10K, ImageNet VID, and Youtube-BB. Images from these datasets were initially preprocessed into image pairs with dimensions of 3 × 127 × 127 as well as 3 × 287 × 287, which are used as template branch (Z) and search branch (X) as the Input. The learning rate was initialized to $5\;\times \;10^{-4}$, decreasing from $10^{-2}$ to $10^{-4}$ in log space. In addition, stochastic gradient descent (SGD) is used with batch size, momentum, and weight decay set to 220, 0.9, and $10^{-4}$, respectively. This tracker was trained on a PC with an Intel i7-11800H CPU, 16 GB RAM, and an NVIDIA GeForce RTX 3060 (8 GB) GPU.

### 4.2. Evaluation Metrics

In this work, we have selected One-Pass Evaluation (OPE) [16] as the assessment method to evaluate the performance of the tracker. The OPE involves two specific metrics: Precision Plot and Success Plot. In the Precision Plot, the horizontal coordinate represents the threshold of the localization error in pixels, while the vertical coordinate represents the ratio of frames where the distance between the centroid of the algorithm’s predicted target frame and the centroid of the real target frame is less than the corresponding threshold, to the total number of frames in the video sequence. As for the Success Plot, it involves setting an Intersection over the Union (IoU) threshold. This plot calculates the ratio of frames in which the IoU between the actual object’s bounding box and the bounding box tracked by the algorithm exceeds the specified threshold relative to the total number of frames in the video sequence.

### 4.3. Ablation Experiment

The ablation experiments in this paper were conducted on the UAV20L dataset. To assess the impact of different modules’ improvements on the experimental results, we present the evaluation results for each set of experiments in Table 1. Specifically, “Ourwork-CFusion” indicates the complete structure with the information interactive fusion module removed during training, “Ourwork-ISA” refers to the complete structure with the sparse enhancement module removed during training, and “Ourwork-MoEn” represents the complete structure trained without the modulation enhancement layer proposed in this work. The symbol “Δ” indicates the change in comparison with “Ourwork”.

As demonstrated in Table 1, the integration of all three innovative modules in this study makes a substantial contribution to optimizing the tracker’s performance. Notably, when the information interactive fusion module is removed from the complete structure during training, there is a noticeable decrease in precision by 9.8% and a decrease in success rate by 8.9%. This result underscores the effectiveness of the proposed interactive fusion of information for small targets, effectively leveraging the information in the overlooked low-level feature maps. Furthermore, the exclusion of both the sparse enhancement module and the modulation enhancement layer from the complete structure during training results in a decline in precision and success rate. This observation confirms the positive effect of incorporating the combination of information from different levels of feature maps, which ultimately enhances the discriminative ability of the features. The outcomes of these ablation experiments provide compelling evidence for the significance of the proposed modules in elevating the overall tracking performance.

### 4.4. Comparision with State-of-the-Art Trackers

The method proposed in this paper is measured on four well-known and authoritative publicly available UAV datasets: UAV123, UAV123@10fps, UAV20L, and DTB70. In this section, the results of this work are shown in comparison with other 36 kinds of SOTA trackers (HiFT [36], DaSiamRPN [42], SiamDW [18], SiamRPN [4], SRDCF [43], MEEM [44], SAMF [45], MUSTER [46], DSST [47], Struck [48], ASLA [49], DCF [50], KCF [50], OAB [51], CSK [52], MOSSE [53], TLD [54], IVT [55], SiamAPN [10], AutoTrack [56], StruckUAV [48], fDSST [47], BACF [57], CoKFC [58], UDT [59], SiamFC [3], ECO [60], MCCT [61], UDT+ [59], TADT [62], ARCF [63], DeepSTRCF [64], C-COT [65], EFSCF [66], SiamRPN++ [5], DSiam [25]).

UAV123: This dataset contains 123 video sequences of more than 112 k frames captured by the UAV on the ground, which contains 12 challenging attributes that the UAV may encounter during the filming of the target.

**Figure 4:**
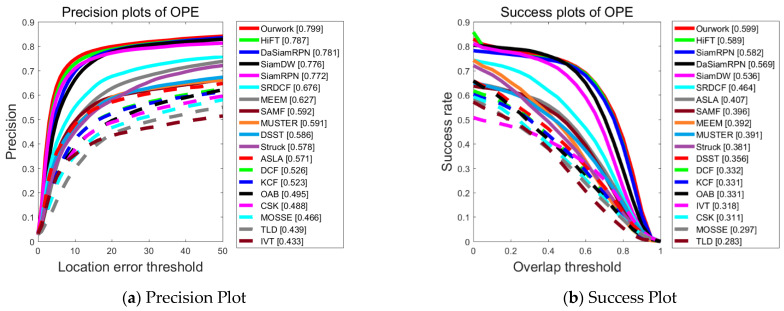
Performance evaluation results of different algorithms on the UAV123 dataset.

As depicted in Figure 4, the proposed tracking algorithm performs significantly better than several other algorithms, such as SiamFC. Compared with the second-ranked network HiFT (ICCV2021), it improves the precision by 1.2% and the success rate by 1%. This indicates that the proposed feature extraction and feature enhancement method, which focuses on using different levels of feature map information, effectively mines and utilizes valuable information within the images. Consequently, it empowers the tracker to excel in completing the tracking task, particularly in the context of UAV-to-ground shooting scenarios.

**Figure 5:**
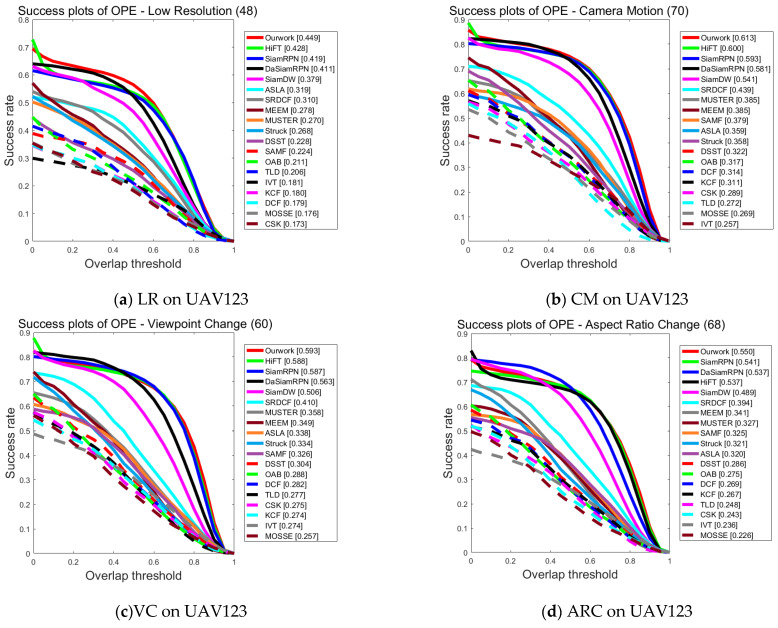

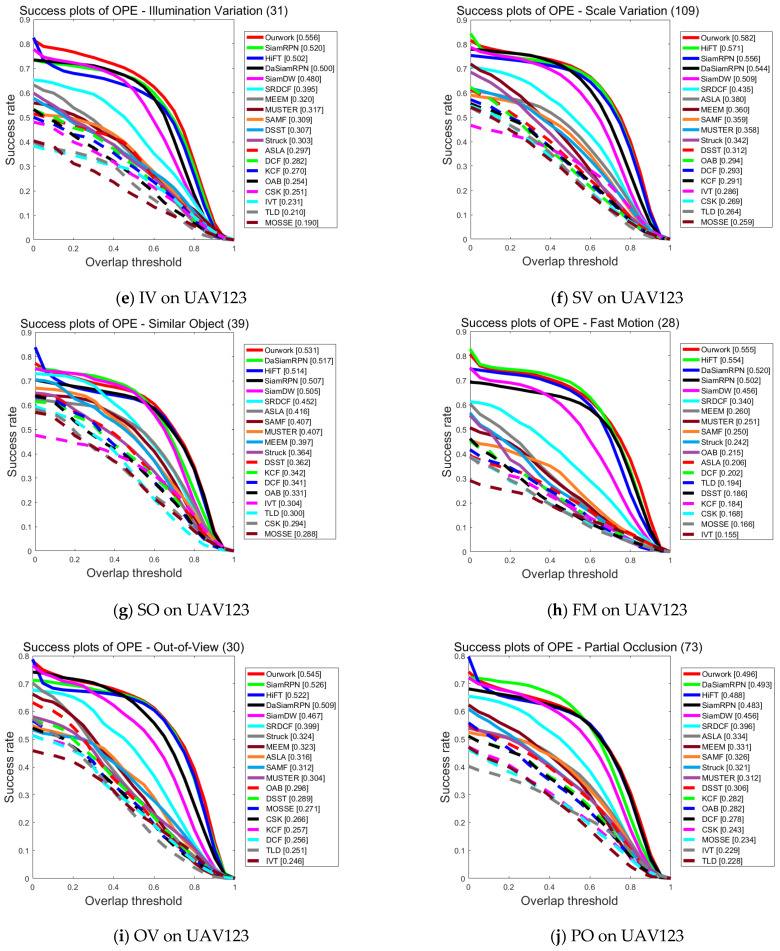

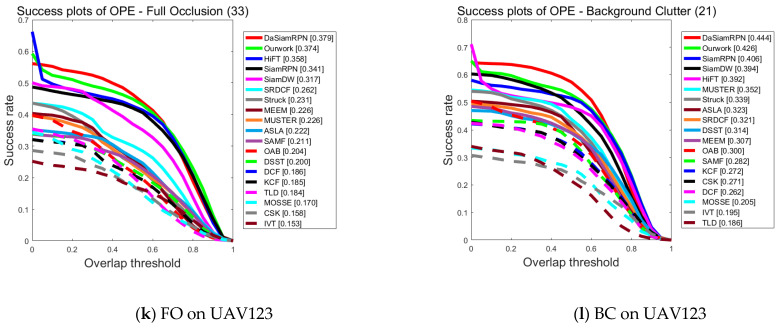
Performance evaluation results of different algorithms on UAV123 for various attributes.

Figure 5a–l provides the detailed results of each tracker on the UAV123 dataset with success maps for each of the 12 attributes, including Low Resolution, Camera Motion, Viewpoint Change, Aspect Ratio Change, Illumination Variation, Scale Variation, Similar Object, Fast Motion, Out-of-View, Partial Occlusion, Full Occlusion, and Background Clutter.

From Figure 5a–l, it can be seen that the tracking algorithm proposed in this paper is ranked first among ten challenges in comparison with 18 other trackers and is able to cope with most of the challenging factors in UAV scenarios. It especially performs well in the challenges of low resolution and camera motion, which confirms that the feature enhancement network proposed in this paper interactively fuses different levels of features and is able to acquire feature information more accurately in the case of low resolution and camera motion. On the other hand, DaSiamRPN primarily addresses object tracking and long-time tracking, lacking the capability for feature information enhancement. Consequently, its performance falls behind that of the algorithm presented in this paper, except for the slightly higher performance in the challenges of full occlusion and background interference, which is the same as that of this paper in all other aspects of the challenge. All in all, the comparison results prove that the algorithm proposed in this work can be adapted to most of the tracking scenarios captured by UAVs.

**Figure 6:**
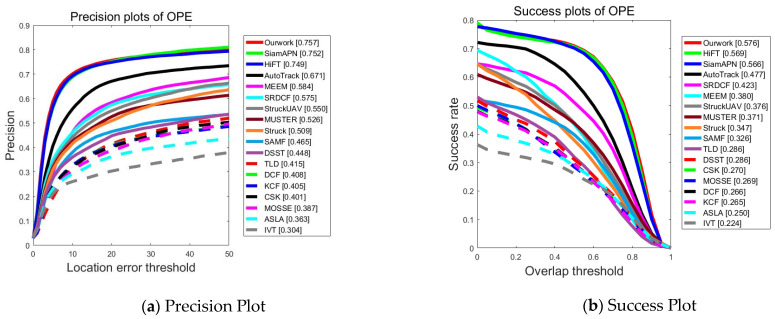
Performance evaluation results of different algorithms on dataset UAV123@10fps dataset.

UAV123@10fps: Created by downsampling the original 30 FPS recording. Consequently, the challenge of strong motion is more severe in UAV123@10fps compared to UAV123. The PPs and SPs, as shown in Figure 6 indicate that the tracker proposed in this work can consistently achieve satisfactory performance with optimal precision (0.757) and success rate (0.576). In summary, it consistently demonstrates more stable performance compared to other SOTA trackers, verifying that it has good robustness in various air tracking scenarios.

DTB70: Contains 70 challenging UAV sequences with a large number of severe motion scenarios. This benchmark can properly evaluate the robustness of the tracker in fast-motion scenarios. The experimental results shown in Table 2 affirm that the tracker proposed in this study secures the top rank in both precision (0.806) and success rate (0.605), with HiFT closely following. The experimental results validate that the tracker in this work is able to cope with the dynamic blurring problem due to fast motion in tracking.

UAV20L: This dataset comprises 20 long videos averaging 2934 frames for a total of 58 K frames. In this paper, it is used to evaluate our tracker in a real long-term aerial tracking scenario. As shown in Table 3, attributed to the enhanced exploitation of global context information by the feature sparsity enhancement module, the tracker proposed in this work achieves competitive performance compared to other SOTA trackers. Specifically, the tracking method proposed in this work yields the best precision score (0.785), outperforming the second-best HiFT (0.763) and the third-best SiamRPN++ (0.696) by 2.2% and 8.9%. Similarly, in terms of success rate, the present working algorithm obtained the best score (0.574), followed by HiFT (0.566) and SiamRPN++ (0.528). The excellent performance underscores the adaptability of the present algorithm to long-time tracking tasks in UAV filming scenarios.

**Figure 7:**
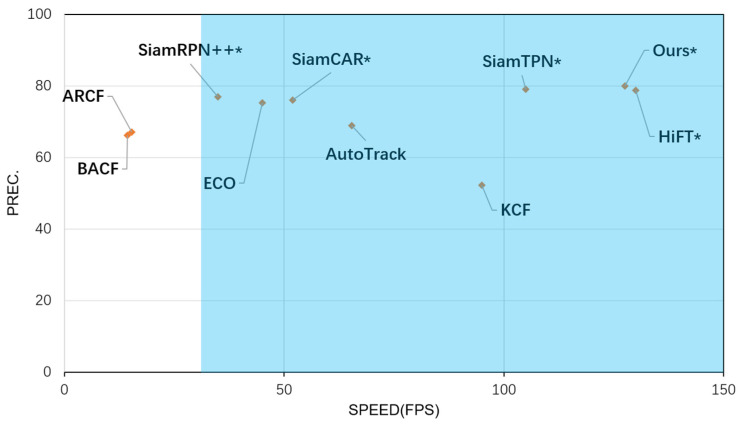
Precision and Speed plot of 10 tracking algorithms on the UAV123 dataset. Note that our results are derived from a PC with an Intel i7-11800H CPU, 16 GB RAM, and an NVIDIA GeForce RTX 3060 (8 GB) GPU. The HiFT’s results are derived from a PC with an Intel i9-9920X CPU, a 32 GB RAM, and two NVIDIA TITAN RTX GPUs.

Finally, as shown in Figure 7, this paper chose to validate the performance of the trackers of this work using a comparative analysis of precision (PREC) versus speed (FPS) tested on the UAV123 dataset with ten trackers. Where trackers marked with * indicate speeds using the GPU, and those not marked with * indicate CPU-only speeds. The blue area indicates that the tracker operation is able to satisfy the real-time, with a speed of more than 30 FPS.

### 4.5. Qualitative Analysis

**Figure 8:**
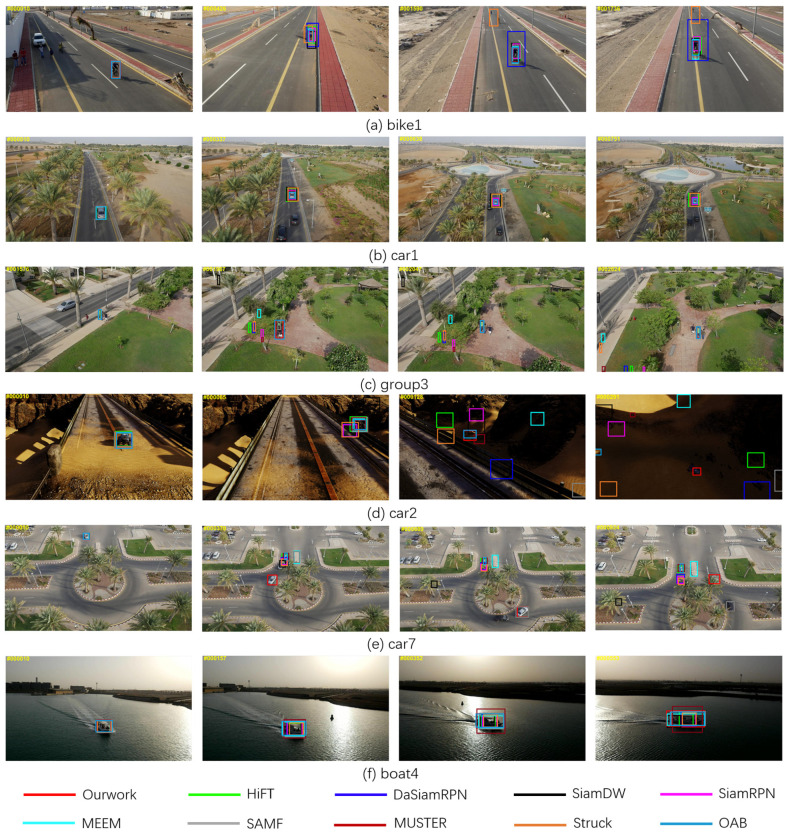
Sample tracking results of evaluated algorithms on different challenging sequences.

To further validate the model’s performance, Figure 8 displays the evaluation results, visualizing the comparison of this work with nine typical tracking algorithms on UAV123, where the comparisons included HiFT [36], DaSiamRPN [42], SiamDW [18], SiamRPN [4], SAMF [45], MEEM [44], MUSTER [46], Struck [48], OAB [51].

As shown in Figure 8a, the bike1 video sequence presents challenges due to a change in viewpoint, which negatively impacts the tracking performance. At the 10th frame, all eight tracking algorithms maintain a good target localization. However, at the 426th frame, SiamRPN, SAMF, and Struck exhibited bounding box shake. By the 1590th and 1736th frames, Struck experienced tracking failure, and SiamRPN showed notably inferior tracking performance.

In Figure 8b, the car1 video sequence encounters challenges related to illumination variation and scale variation. In the later stages of tracking, HiFT, SAMF, and Struck trackers demonstrate inferior performance compared to the tracking algorithm proposed in this work.

Figure 8c depicts the group3 video sequence, which contains challenges such as interference from similar objects and partial occlusion. Leveraging the comprehensive utilization of features from different layers, our approach continues to accurately track the target, demonstrating the robustness of the tracking algorithm.

In Figure 8d, the tracking encounters light and shadow variations as well as low resolution. It can be seen that only the method proposed in this paper is successful in tracking in frames 128 and 291.

In Figure 8e, the car7 scenario presents a situation where, in the 370th frame, tracking encounters difficulties such as partial occlusion and interference from similar objects. Notably, all other tracking methods fail in this scenario except for the algorithm proposed in this paper. Although SAMF manages to re-establish tracking in the 689th frame, it experiences another tracking failure in the 924th frame.

In Figure 8f, except for our method, the other nine trackers are not well adapted to the challenge of changing the aspect ratio of the object tracking during tracking. The tracker’s performance across video sequences with diverse challenges serves as compelling evidence, affirming the robustness of the tracker introduced in this paper. This reaffirms its ability to consistently excel in tracking tasks, even under challenging conditions.

## 5. Conclusions

Addressing the challenges posed by low-resolution UAV tracking targets and the potential for dynamic blurring due to fast motion, this paper proposes a tracker that combines information interactive fusion and hierarchical Transformer. The lightweight network, AlexNet, is employed as the backbone network to extract five layers of features from the template image and the search image, and the interactive fusion module is used to interactively fuse the last three layers of features in order to mine the useful information in the low-level feature maps that are neglected by ordinary trackers. After sparse enhancement of the third layer, which contains the most information, the obtained three-layer feature maps are hierarchically fed into the Transformer to enhance the tracker’s exploitation of the global contextual information and to extract the dependencies between different layers and exploit them in the encoder. The experimental results demonstrate that the tracking algorithm proposed in this work improves the tracking precision and success rates compared to existing conventional trackers. It effectively adapts to the challenges posed by small object tracking, low pixel resolution, and dynamic blur scenarios in UAV tracking.

## Figures and Tables

**Table 1:** Experimental results of different modules on the UAV20L dataset.

Trackers	Precision	$\Delta_{pre}$(%)	Success	$\Delta_{suc}$(%)
Ourwork	0.785	-	0.574	-
Ourwork-CFusion	0.687	−9.8	0.485	−8.9
Ourwork-ISA	0.734	−5.1	0.526	−4.8
Ourwork-MoEn	0.712	−7.3	0.509	−6.5

**Table 2:** Overall evaluation on DTB70. Prec. And Succ. Denote precision score at 20 pixels and AUC of success plot.

Trackers	Prec.	Succ.	Trackers	Prec.	Succ.
fDSST	0.534	0.357	AutoTrack	0.716	0.478
BACF	0.590	0.402	SiamFC	0.719	0.483
CoKFC	0.599	0.378	ECO	0.722	0.502
UDT	0.602	0.422	MCCT	0.725	0.484
STRCF	0.649	0.437	DeepSTRCF	0.734	0.506
UDT+	0.658	0.462	C-COT	0.769	0.517
TADT	0.693	0.464	SiamRPN++	0.795	0.589
DaSiamRPN	0.694	0.472	HiFT	0.802	0.594
ARCF	0.694	0.472	**Ours**	**0.806**	**0.605**

**Table 3:** Overall evaluation on UAV20L, Prec. And Succ. Denote precision score at 20 pixels and AUC of success plot.

Tracker	ECO	DeepSTRCF	SiameseFC	Dsiam	EFSCF	DaSiamRPN	SiamRPN++	HiFT	Ours
Prec.	0.589	0.588	0.599	0.603	0.604	0.665	0.696	0.763	**0.785**
Succ.	0.427	0.443	0.402	0.391	0.452	0.465	0.528	0.566	**0.574**

## Data Availability

Data will be made available on request.
